# Common Barriers to Reporting Medical Errors

**DOI:** 10.1155/2021/6494889

**Published:** 2021-06-10

**Authors:** Salim Aljabari, Zuhal Kadhim

**Affiliations:** ^**1**^ Child Health Department, University of Missouri-Columbia, Columbia, MO, USA; ^**2**^ Department of Family and Community Medicine, University of Missouri-Columbia, Columbia, MO, USA

## Abstract

**Background:**

Medical errors are the third leading cause of death in the United States. Reporting of all medical errors is important to better understand the problem and to implement solutions based on root causes. Underreporting of medical errors is a common and a challenging obstacle in the fight for patient safety. The goal of this study is to review common barriers to reporting medical errors.

**Methods:**

We systematically reviewed the literature by searching the MEDLINE and SCOPUS databases for studies on barriers to reporting medical errors. The preferred reporting items for systematic reviews and meta-analyses guideline was followed in selecting eligible studies.

**Results:**

Thirty studies were included in the final review, 8 of which were from the United States. The majority of the studies used self-administered questionnaires (75%) to collect data. Nurses were the most studied providers (87%), followed by physicians (27%). Fear of consequences is the most reported barrier (63%), followed by lack of feedback (27%) and work climate/culture (27%). Barriers to reporting were highly variable between different centers.

## 1. Introduction

Medical errors (ME) are among the most important patient safety challenges facing hospitals and healthcare systems nowadays. Since the Institute of Medicine (IOM) report in 1999 “To Err is Human,” an increasing number of studies have shown how prevalent and deleterious ME are, especially in hospital medicine [1]. With this, healthcare leaders invested time and resources toward identifying and reducing ME [2].

A medical error is defined as “an incidence when there is an omission or commission in planning or execution that leads or could lead to unintended result” [3]. While the majority of ME do not lead to an apparent adverse effect, a significant number of patients either suffer a permanent injury or death from ME every year in the United States and around the world as a result of those errors [4].

Medical errors are the third leading cause of death in the United States after heart diseases and cancer [4]. It is estimated that more than 200,000 patients die annually in the United States from ME [5]. Furthermore, in addition to the harm inflicted on patients, medical errors are associated with an increased healthcare cost [6]. In a 2008 report, it was estimated that medical errors costed the healthcare system in the United States more than 17 billion dollars annually [7, 8].

The first step in combating ME and improving patient safety is to study the different types of medical errors to better understand why medical errors happen. The causes, types, and rates of ME can vary from one institution to the other and change over time, especially as we implement changes in our healthcare delivery. Therefore, it is important to capture, track, and analyze all medical errors as possible at the institutional level [2, 9, 10].

As most of the nonmedication medical errors are hard to capture electronically and manual chart review is both cumbersome and time consuming, self-reporting is still the most reliable approach to capture ME [11]. Unfortunately, underreporting of ME is a commonly reported challenge even when healthcare institutions mandated reporting [12]. While there is no consensus on what defines “underreporting of ME,” it commonly refers to the lack of reports on significant ME events. The goal of this study is to review the reported perceived barriers to reporting medical errors by healthcare providers in hospital settings and to identify common themes.

Most of the reports on barriers to reporting ME are single centers; in this systematic review of the literature, we try to investigate whether the barriers to reporting ME varies from institution to the other or not and what common barriers are reported.

## 2. Methodology

We conducted a systemic review in accordance with the preferred reporting items for systematic reviews and meta-analyses (PRISMA) guideline [13].

### 2.1. Data Sources and Search Strategy

We queried MEDLINE (2000–2020) and SCOPUS (2000–2020) databases for eligible studies. The year 2000 was chosen as the start date for eligibility as the vast of majority of publications regarding ME came after the IOM report in 1999 [1].

On MEDLLINE, a combination of the following search terms was used: (i) errors (medical subject heading (MeSH)), medication errors (MeSH) or near mess, and healthcare (MeSH), (ii) hospitals (MeSH), and (iii) disclosure (MeSH), “report$” (in the title), “ident$” (in the title), or “recog$” (in the title).

On Scopus, the following search string was used: (TITLE ((medica^*∗*^ AND error) AND (report^*∗*^ OR captur^*∗*^)) AND (LIMIT-TO (PUBSTAGE, “final”)) AND (LIMIT-TO (DOCTYPE, “ar”)) AND (LIMIT-TO (LANGUAGE, “English”))).

We manually removed duplicate studies, and we also evaluated additional eligible studies in the references of the final selected studies.

### 2.2. Study Selection and Data Extraction

**Figure 1:**
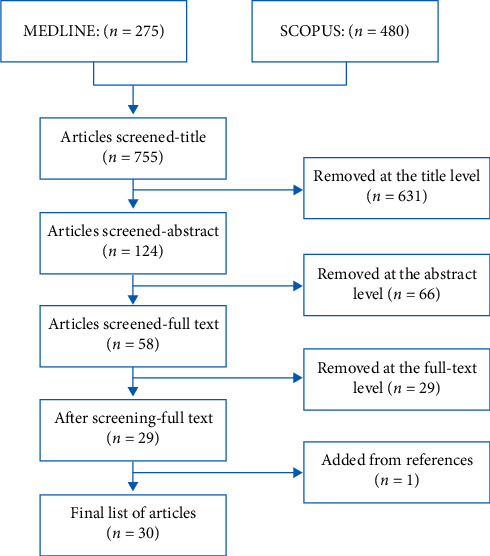
The study selection process using the PRISMA guideline.

The returned studies were evaluated for proper content. Studies were screened for the following inclusion criteria: (i) English language, (ii) the focus of the research is to identify barriers to ME reporting, (iii) medical errors as defined above, not diagnostic errors or management errors, (iv) in hospital settings, and (v) full-text articles. The overview of the selection process is summarized in Figure 1. The primary investigator screened the citations from the initial search using two-step approach. First, the titles and abstracts of all selected articles were screened for eligibility. Then, for the citations that considered relevant, the full-text we obtained was screened for eligibility.

The following data elements were extracted from the final list of eligible studies: primary objective, study design, sample size, study setting, study subjects, country of the study, year of publication, recruitment of subjects, response rate in survey studies, pertinent results, primary outcomes, and limitations of the study.

## 3. Results

The search yielded 755 studies of which 30 studies met the inclusion criteria. Figure 1 highlights the studies selection process.  Table 1 is a brief summary of the included studies [14–43]. Eight of the selected studies were from the United States. The majority of the studies (74%) used self-administered questionnaires to identify perceived barriers for ME reporting. Three studies did post hoc analysis of national databases; those national databases were the results of self-administered questionnaires.

**Figure 2:**
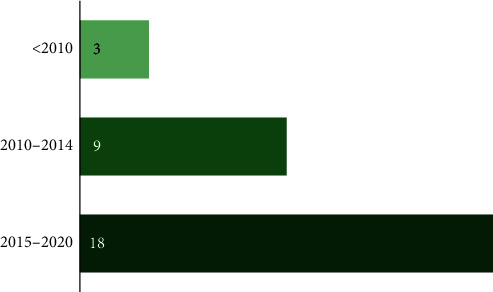
Year of publication of the selected studies.

As shown in Figure 2, most of the included studies are relatively recent. Nurses were the most surveyed/studied healthcare providers, included in 26 (87%) included studies, followed by physicians (27%) and pharmacists (17%). Some of the studies (23%) recruited subjects from specific inpatient units, and the rest recruited subjects from all inpatient units. Eighteen of the included studies evaluated perceived barriers to reporting medication errors or medication administration errors, and the rest evaluated perceived barriers to reporting any medical error which included medication errors.

### 3.1. Barriers to Reporting ME

We identified 7 common themes to the barriers reported in the included studies (Table 2). We discuss the common themes in the following sections.

#### 3.1.1. Fear of Consequences

Fear of consequences is the most reported factor for underreporting ME. 19 out of the 30 studies reported that fear is a significant barrier to report ME [14–16, 20–22, 25, 37–40, 42].

Fear of being blamed for the error is by far the most reported fear. But additionally, providers reported fear of losing one's job, fear of patient's or family's response to the ME, fear of being recognized as incompetent, fear of legal consequences, fear of punishment, and fear of losing respect by coworkers were also commonly reported [14–16, 20–22, 25, 32, 37–40, 42].

**Figure 3:**
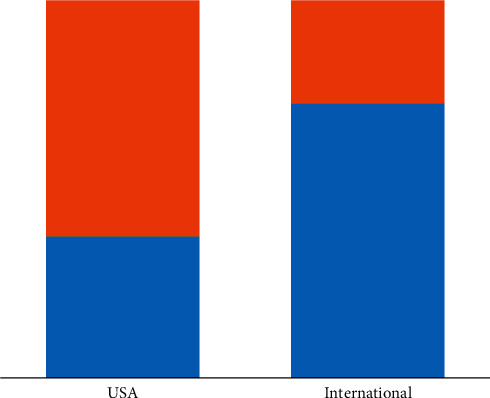
Percentage of studies where “fear of consequences” is an important barrier. Blue, yes, “fear of consequences” is an important barrier. Orange, no, “fear of consequences” is not an important barrier.

Not only is “fear of consequences” the most reported factor for underreporting, it also happens to be the most significant factor for underreporting in most of the included studies [14–16, 20–22, 25, 32, 37–40, 42]. While fear of consequences might be more prominent in certain cultures than others and more prominent in hospitals with hierarchical structures [16], it has been reported at both local and international levels and in different management styles. Additionally, fear as a factor has not changed over the years. It is unclear whether an option to anonymously report ME would eliminate the fear barrier [14–16, 20–22, 25, 32, 37–40, 42]. It does, however, seem that “fear of consequences” as a barrier to reporting is less prevalent in the United States compared to other countries (Figure 3).

It is important to highlight that some of the included studies did not find “fear of consequences” as a significant factor for underreporting [41]. Findings from those studies suggest that we can overcome “fear of consequences” as a barrier.

#### 3.1.2. Lack of Feedback

Both lack of feedback by administration and/or negative feedback have been associated with underreporting. While some studies reported the negative impact of improper feedback, some reported the positive impact of appropriate feedback. Specifically, it was evident that feedback to the reporting person about the error supports the provider who committed the error and communication openness regarding errors all improved reporting of ME [14, 22, 27, 29, 31, 36, 40–42].

#### 3.1.3. Work Climate/Culture

The administration's attitude toward ME and the work environment are important factors that influence ME reporting [17, 21, 40, 42]. It has been observed that when hospital administrators' responses to ME focus on the individuals, rather than the system, reporting rates of ME decrease [21]. Additionally, the lack of safety culture and error prevention programs is associated with underreporting [27]. On the other hand, work environments with a strong teamwork perception and psychological safety amongst employees are associated with better reporting of ME [30, 32]. Work climate/culture issues as a barrier to reporting medical errors is the most reported barrier in studies from the United States (Table 1).

#### 3.1.4. Poor Understanding of ME and the Importance of Reporting ME

A number of studies reported poor understanding by providers as to what constitute a medical error as a barrier to reporting. Providers in a number of studies reported the lack of clear definition of medical errors and the lack of a clear protocol on what incidents need to be reported, as a significant barrier to reporting ME. Additionally, poor understanding of the importance of reporting ME is a significant barrier to reporting as well [21, 23, 35, 38, 41, 44].

#### 3.1.5. Time Consuming

Busy work schedule and high workload have been reported as significant factors for underreporting. Additionally, reporting itself is time consuming and cumbersome. Both forms of reporting systems (paper and electronic) are time consuming [20, 23, 25, 38, 41, 44]. Physicians more than nurses reported time constrains as a barrier to reporting ME [41].

#### 3.1.6. Lack of the Reporting System

It is no surprise that the lack of a reporting system is a barrier. Many studies, mostly international, reported the lack of a reporting system as a barrier to reporting [22, 29, 35]. A number of studies showed better reporting with electronic systems compared to paper reporting [45].

#### 3.1.7. Personal Factors

A number of personal factors influence the reporting of ME. Younger and/or less experienced nurses are less likely to report ME. The longer the employment period is, the more likely it is for an employee to report ME. Additionally, personal experience with ME affected the rates of reporting medical errors [17, 36, 40, 42].

## 4. Discussion

In this systematic review of literature, we present reported barriers to ME reporting in hospital setting. We identified and presented common themes to the reported barriers. We also highlighted the variation in perceived barriers between different centers and countries.

The healthcare system and healthcare delivery vary from one country to the other. Thus, it is no surprise that perceived barriers to reporting were also variable between different countries. For example, “fear of consequences” is more prevalent in East Asia and Middle East compared to the United States. On the other hand, work climate/culture is more reported as barrier in centers across the United States. Reported barriers also varied from one center to the other within the same country. These differences are probably secondary to different management strategies, reporting systems, different work place culture, and whether patient safety is a focus of the hospital administration or not.

Nurses, physicians, and pharmacists are the most studied groups of providers regarding ME and the reporting of ME. Unfortunately, none of the studies directly compared the barriers perceived by these different groups. It is logical to anticipate different perception of barriers between these groups of the provider. Additionally, current studies failed to include other groups of clinical providers such as respiratory therapists, physical/occupational therapists, and laboratory and radiology technicians, despite their significant role in hospital medicine.

Fear of consequences is reported in most of the studies we reviewed as one of the important barriers to reporting ME. Some of the sources for fear are modifiable, for example, fear of being blamed for the error or fear of losing one's job. Changing workplace culture and strategies in addressing reporting ME is an imperative step to overcome this barrier. A work culture that promotes patient safety, encourages error reporting, and implements system changes is essential. On the other hand, fear secondary to concern over patients' and their families' reactions to medical error is not modifiable or predictable. Educating the providers on the importance of ME disclosure to the patients/families and providing them with the necessary tools to better communicate ME and adverse events can help overcome some of these nonmodifiable fears.

The most challenging and probably most effective change to overcome barriers to reporting medical errors is the adoption of patient safety culture. Under patient safety culture, employees are rewarded and feel empowered to report and act on medical errors. This safety culture helps overcome the employee's fear of consequences and provides a work environment that is supportive of error recognition and reporting.

The reviewed studies showed that a significant number of healthcare providers lack proper understanding of what constitutes a medical error. Poor understanding of medical errors and the importance of reporting both lead to underreporting. Educating healthcare providers on what constitutes medical errors, the benefit of reporting medical errors even in the absence of apparent harm, and that medical error reports are used to identify system deficiencies rather than individual faults, can help improve ME reporting and eventually decrease ME.

As hospitals across the world are adopting changes in their management and care delivery to improve patient's safety, the barriers to reporting medical errors may change. Periodic evaluation of this matter is needed to continue the improvement process.

Healthcare institutions are encouraged to evaluate their ME reporting rates, perform root cause analysis for underreporting at the local level, and finally implement changes to improve reporting. The common themes we identified in this study can guide healthcare institutions in their local root cause analysis. Causes of ME and factors for underreporting ME may change with time as we implement changes to our healthcare delivery. Thus, continuous tracking of ME and periodic evaluation of the root causes is needed to continue the improvement process. In some institutions, deep changes in the hospital's management strategy to align with and encourage patient safety culture might be needed.

Our study has several limitations. The first limitation is inherent to the nature of survey and interview studies. All published reports on this matter used either self-administered questionnaires or interviews. The second limitation is inherent to the nature of systematic review of the literature. The variability of study methodology and study population makes it challenging to draw an objective conclusion. Due to the variability in the methodology and study population in the selected studies, a meta-analysis is not feasible and only a subjective conclusion can be presented.

## 5. Conclusion

We identified and presented 7 common themes of barriers to report medical errors. Fear of consequences is the most reported barrier worldwide, while work climate/culture is the most reported barrier in the United States. Barriers to reporting can vary from one center to the other. Each healthcare institution should identify local barriers to reporting and implement potential solutions. Overcoming the barriers may require changes in the hospital's management strategy to align with and encourage a patient safety culture. Further studies are needed to investigate whether an anonymous reporting system can help overcome the fear barrier and to compare perceived barriers to report ME between different healthcare providers.

## Figures and Tables

**Table 1:** Summary of the selected studies.

Reference	Country	Publication year	Objective	Study design	Sample size (response rate, %)	Subjects	Setting	Results (most important barriers reported by themes)
Morrison et al. [12]	Iran	2020	Medical errors	Survey	164 (78)	Nurses (*n* = 77) Physicians (*n* = 87)	Single hospital	Fear of consequences
								Lack of feedback
Moher et al. [13]	Turkey	2019	Medication errors	Survey	135 (53)	Nurses	Single hospital	Fear of consequences
								Poo understanding of ME and importance of reporting
Mahdaviazad et al. [14]	South Korea	2019	Medication errors	Survey	218 (81)	Nurses	Multicenter (7 hospitals)	Fear of consequences
Dirik et al. [15]	Iran	2019	Medical errors	Interviews	18 (NA)	Nurses	Unit specific (pediatric inpatient)	Fear of consequences
								Work climate/culture
Kim and Kim [16]	USA	2018	Medication errors	Survey	357 (36)	Nurses	Single hospital	Time consuming
								Fear of consequences
Mousavi-Roknabadi et al. [17]	Qatar	2018	Medication errors	Survey	1604 (NA)	Nurses (*n* = 1089) Physicians (*n* = 213) Pharmacists (*n* = 207)	Single hospital	Fear of consequences
Rutledge et al. [18]	Iran	2017	Medication errors	Survey	328 (65)	Nurses	Multicenter (7 hospitals)	Time consuming
								Fear of consequences
Stewart et al. [19]	Saudi	2017	Medication errors	Survey	367 (73)	Nurses	Single hospital	Fear of consequences
Fathi et al. [20]	Turkey	2017	Medical errors	Interviews	23 (NA)	Nurses (*n* = 15) Physicians (*n* = 8)	Single hospital	Fear of consequences
								Lack of feedback
								Lack of a reporting system
Hammoudi and Yahya [21]	South Korea	2017	Medication errors	Survey	245 (33)	Pharmacists	Multicenter (32 hospitals)	Lack of understanding of ME and the importance of reporting ME
Soydemir et al. [22]	Iran	2017	Medical errors	Survey	140 (NA)	Nurses	Unit specific (obstetric ward)	Fear of consequences
Kang et al. [23]	USA	2017	Medication errors	Survey	71 (45)	Nurses	Unit specific (ER)	Lack of feedback
								Work climate/culture factors
Mobarakabadi et al. [24]	UAE	2016	Medication errors	Interviews	29 (NA)	Nurses (*n* = 10) Pharmacists (*n* = 10) Physicians (*n* = 9)	Multicenter (3 hospitals)	Lack of feedback
								Work climate/culture
Farag et al. [25]	USA	2016	Medical errors	Post hoc analysis of a national database	5339 (NA)	Pharmacists	Multicenter (NA)	Lack of feedback
								Work climate/culture
Alqubaisi et al. [26]	Taiwan	2016	Medication errors	Survey	15 (41)	Nurses	Single hospital	Fear from consequences
Patterson and Pace [27]	Iran	2015	Medical errors	Survey	348 (16)	Physicians, nurses, and others	Multicenter (5 hospitals)	Lack of a reporting system
								Lack of understanding of ME and the importance of reporting ME
Yung et al. [28]	USA	2015	Medical errors	Post hoc analysis of a national database	NA	All employees	Multicenter (NA)	Work climate/culture
Poorolajal et al. [29]	USA	2015	Medical errors	Survey	40 (60%)	Nurses	Unit specific (surgical)	Work climate/culture
Derickson et al. [30]	USA	2014	Medical errors	Survey	300 (75)	Nurses (*n* = 186) Physicians (*n* = 26) Paramedics (*n* = 78)	Single hospital	Fear of consequences
Farag and Anthony [31]	South Korea	2014	Medical	Survey	522 (77)	Nurses	Multicenter (2 hospitals)	Work climate/culture
Jahromi et al. [32]	UK	2014	Medication errors	Interviews	50 (NA)	Nurses	Unit specific (psychiatric hospital)	Time consuming
								Lack of understanding of ME and the importance of reporting ME
								Fear of consequences
Hwang and Ahn [33]	Iran	2014	Medication errors	Survey	100 (NA)	Nurses	Single hospital	Lack of a reporting system
								Lack of feedback
								Lack of understanding of ME and the importance of reporting ME
Haw et al. [34]	USA	2013	Medical errors	Post hoc analysis of a national database	5339 (NA)	Pharmacists	Multicenter (NA)	Work climate/culture
Mostafaei et al. [35]	Saudi	2013	Medication errors	Survey	307 (88)	Nurses	Single hospital	Fear of consequences
Patterson et al. [36]	Canada	2012	Medication errors	Focus group	NA	NA	Multicenter (4 hospitals)	Time consuming
								Fear of consequences
Aboshaiqah [37]	Turkey	2012	Medication errors	Survey	119 (72)	Nurses	Unit specific (pediatrics)	Fear of consequences
Hartnell et al. [38]	Taiwan	2010	Medication errors	Survey	838 (84)	Nurses	Multicenter (5 hospitals)	Fear of consequences
Toruner and Uysal [39]	UK	2009	Medical errors	Survey	134 (66)	Nurses (*n* = 82) Physicians (*n* = 55)	Unit specific (surgical units)	Lack of understanding of ME and the importance of reporting ME
								Lack of feedback
Chiang [40]	Taiwan	2006	Medication errors	Survey	597 (74)	Nurses	Single hospital	Fear of consequences
								Time consuming
								Lack of feedback
Kreckler et al. [41]	USA	2005	Medication errors	Survey	25 (41)	Nurses	Single hospital	Fear of consequences

**Table 2:** Common themes of barriers to reporting medical errors.

Theme	Number of studies reported this theme as a significant barrier
Fear of consequences	19
Lack of feedback	8
Work climate/culture	8
Poor understanding of ME and the importance of reporting ME	6
Time consuming	5
Lack of a reporting system	3
Personal factors	3
